# Auswirkungen der Covid-19-Pandemie auf den Einsatz von Kreativitätstechniken: Herausforderungen, Lösungsansätze und Modelle für die Online-Kommunikation in Virtuellen Teams

**DOI:** 10.1365/s40702-021-00752-w

**Published:** 2021-06-28

**Authors:** Klemens Waldhör, Leonie Kubla

**Affiliations:** FOM Hochschule für Oekonomie & Management, Hochschulzentrum Nürnberg, City Park Center, Zeltnerstr. 19, 90443 Nürnberg, Deutschland

**Keywords:** Kreativitätstechniken, Covid-19 Pandemie, Online-Kommunikation, Evaluation, Creativity techniques, Covid-19 Pandemia, Online-communication, Evaluation

## Abstract

In diesem Beitrag sollen die Veränderungen beim Einsatz von Kreativitätstechniken in der aktuellen Covid-19-Pandemie untersucht werden. Basierend auf einer Evaluationsmatrix und einem Entscheidungsmodell wird eine Liste von Kreativitätstechniken ermittelt und bewertet, die online erfolgreich in virtuellen Teams oder im Home Office verwendet werden können. Eine wichtige Anforderung war, dass die ermittelten Kreativitätstechniken ohne größeren Aufwand oder Neuimplementierung in Online-Umgebungen wie Zoom, Microsoft Teams und in verwandten Systemen eingesetzt werden können. Damit gibt der Beitrag eine Unterstützungs- und Entscheidungshilfe, wie in Pandemiezeiten die Kreativität der Mitarbeiter in Online-Arbeitsumgebungen gefördert werden und Teams trotz der Einschränkungen gemeinsame Ideen und innovative Produkte und Anwendungen entwickeln können. Die Erkenntnisse und Modelle sind nicht nur für den Softwarebereich geeignet, sondern für alle Anwendungsbereiche, die sich mit der Entwicklung innovativer Produkte beschäftigen. Ebenso können sie im schulischen und universitären Bereich verwendet werden.

**Zusatzmaterial online:**

Zusätzliche Informationen sind in der Online-Version dieses Artikels (10.1365/s40702-021-00752-w) enthalten.

## Abstract (en)

The purpose of this paper is to examine the changes in the use of creativity techniques in the current Covid 19 pandemic. Based on an evaluation matrix and a decision model, a list of creativity techniques that can be successfully used online in virtual teams or home offices is identified and evaluated. An important requirement was that the identified creativity techniques can be used in online environments such as Zoom, Microsoft Teams, and in related systems without significant effort or re-implementation. Thus, the paper provides support and decision support on how to foster employee creativity in online work environments during pandemic times and how teams can develop collaborative ideas and innovative products and applications despite the given constraints. The findings and models are not only suitable for the software domain, but for all application areas that deal with the development of innovative products. Likewise, they can be used in the school and university sector.

## Problemstellung und Motivation

Die Covid-19-Pandemie hat innerhalb kurzer Zeit große Teile der Arbeitswelt in einer Weise verändert, wie man es vor einem Jahr nicht erwartet hätte. Lockdownbasierte Schließungen ganzer Unternehmen und die damit verbundene Verlagerung der täglichen Arbeit nach Hause haben massive Folgen für die Wirtschaft und die einzelnen Mitarbeiter. Home Office ist neben Distance Learning für Schulen eines der am meisten diskutierten und umstrittenen Themen der Jahre 2020 und 2021. Physische Teams haben sich in kurzer Zeit in virtuelle Teams verwandelt. Damit haben sich auch die Anforderungen an Teamarbeit und Teamführung verändert.

Welche Auswirkungen hat dies aber auf die Softwareindustrie? Sind diese ebenso gravierend wie für andere Industriebereiche? Oder sind die Auswirkungen nicht so gravierend, weil die Produktion von Software ohnehin schon seit Längerem eine arbeitsteilige Tätigkeit ist, die leicht auslagerbar ist und örtlich basierte Zusammenarbeit weniger wichtig ist?

In diesem Beitrag wird nicht der allgemeine Aspekt dieser Fragen betrachtet, sondern die Situation speziell in Hinblick auf Kreativitätstechniken und verwandte Techniken.

## Rolle der Kreativitätstechniken im Softwareentwicklungsprozess

Die Definition des geplanten Produktes oder Artefaktes, das den Kundenwünschen optimal entspricht, stellt einen Kernpunkt in den frühen Aktivitäten in Softwareprojekten dar. Betrachtet man das Kano-Modell (Sauerwein 2000), so führen insbesondere solche Produkteigenschaften (Begeisterungsmerkmale) zur Kundenzufriedenheit, die a) einen wirklichen Mehrwert bieten und b) vom Kunden nicht erwartet werden, ihn überraschen. Diese beiden Eigenschaften erfordern aber einerseits eine intensive Beschäftigung mit dem Kunden und seinen Wünschen, andererseits innovative Ideen und entsprechende Kreativität beim Entwurf des Artefaktes. Sie erfordern einerseits ein tiefes Wissen über die Kundenbedürfnisse und des Marktes, andererseits ein entsprechendes Mindset wie Offenheit für Neues und Kundenorientierung (Mezick et al. 2019; Guttenberger 2018; Dark Horse Innovation 2017). In der Softwareentwicklung hat sich seit den 90er-Jahren die Erkenntnis durchgesetzt, dass Programmierer meist weniger gut im Design eines Produkts sind. Besser geeignet sind Teams mit unterschiedlichen Spezialisten (Raskin 2001; Johnson 1999; Norman 1999). Eine fundamentale Erkenntnis ist, dass die Involvierung des Anwenders und eine vertrauensvolle Kommunikation mit ihm und im Team in den einzelnen Projektphasen Fehlschläge vermeiden, was aus den Grundprinzipien des agilen Manifests (Agile Alliance 2001) folgt.

Im klassischen Vorpandemie-Szenario werden Produktaspekte gemeinsam mit dem Kunden neben anderen aus dem Softwareengineering bekannten Techniken unter Einsatz von Kreativitätstechniken physisch vor Ort erarbeitet. Die überwiegende Anzahl der bekannten Kreativitätstechniken, aber auch Team-orientierter Problemlösungstechniken, basieren auf diesem Vor-Ort-Prinzip. Die Teilnehmer treffen sich persönlich in einem Seminarraum und entwickeln die Grundideen des Artefakts. Diese Unmittelbarkeit des Produktentstehungserlebnisses für den Kunden betonen auch die modernen agilen Methoden und Ansätze wie beispielweise Design Thinking (Brenner und Uebernickel 2016; Plattner et al. 2016). Die physische Präsenz erhöht die Zufriedenheit und Akzeptanz durch den Kunden und vermeidet Fehlentwicklungen. Die Pandemie hat die Situation grundlegend geändert. Persönliche Treffen waren und sind aus Gesundheitsgründen nicht mehr möglich oder erwünscht, Kunden vermeiden den persönlichen Kontakt, die Arbeit wird in das Home Office verlegt.

## Ziele

Dieser Beitrag hat als Ziel zu eruieren, welche Kreativitätstechniken in einer solchen stark durch Online-Kommunikation geprägten Umgebung sinnvoll einsetzbar sind. Die von Unternehmen eingesetzten Softwarelösungen wie Microsoft Teams, Zoom oder ähnliche sollen ohne größere Änderungen weiter verwendet werden können. Dazu wird eine Evaluationsmatrix entwickelt, die die wesentlichen Kreativitätstechniken anhand verschiedener Merkmale charakterisiert. Basierend darauf wird ein Entscheidungsmodell vorgestellt, das es Teams ermöglicht Kreativitätstechniken auf ihre Eignung in der Online-Kommunikation zu bewerten. Dabei wird als Online-Kommunikation die Kommunikation über Webkonferenzsysteme verstanden, die die Funktionen der Audio- und Videoübertragung, des Chats und der Bildschirmübertragung beinhalten.

## Theoretische Grundlagen der Online-Kommunikation und Kreativität

In diesem Beitrag wird *Online-Kommunikation* als menschliche Kommunikation, die online in Gruppen stattfindet, verstanden. Das zur Kommunikation genutzte Medium ist somit das Internet, das neue räumliche und zeitliche Kommunikationsmöglichkeiten eröffnet (Fraas et al. 2012). Da die Kommunikation per Videokonferenz der Face-to-Face-Kommunikation, die die persönlichste und intensivste Form der Kommunikation darstellt, am nächsten kommt, werden hier insbesondere Webkonferenzsysteme als die Kommunikationsform betrachtet (Beck 2020).

Als *Gruppe *gelten zwei oder mehr Individuen, die durch soziale Beziehungen miteinander verbunden sind (Forsyth et al. 2019). Nach Rosenstiel (2003) und Spieß und Rosenstiel (2010) weist eine Gruppe Merkmale wie mehrere Personen, verschiedene Rollen, gemeinsame Ziele, Werte, Normen und Zusammengehörigkeitsgefühl sowie eine Interaktion über einen längeren Zeitraum auf. Der Begriff des *Teams* wird häufig als Synonym für Gruppe gebraucht. In diesem Beitrag wird Team als Sonderform einer Gruppe angesehen, das durch ein gemeinsames Ziel miteinander verbunden ist (Forsyth et al. 2019). Mit dem Begriff Team ist zumeist eine positive Konnotation verbunden, während Gruppe auch negativ konnotiert sein kann. Teams können in *Collocated Teams* (Kommunikation meist ohne elektronisches Material und in persönlichen Treffen), *Virtuelle Teams* (Kommunikation hauptsächlich elektronisch, eher gleichrangige Teammitglieder, breitere Streuung der Tätigkeitsfelder über die Orte) und *Remote Teams* (Häufig keine gleichrangigen Teammitglieder, keine breite Streuung der Tätigkeitsfelder über die Orte, Auslagerung des Programmierteams auf entfernten Standort, Steuerung durch lokale Teamleiter und Mitarbeiter, klare ortsgebundene Arbeitsteilung) klassifiziert werden.

Die Literatur verwendet unterschiedliche Definitionen für *Kreativität*. Drevdahl definierte 1956 Kreativität beispielsweise als „die Fähigkeit von Personen Kompositionen, Produkte oder Ideen jeglicher Art zu produzieren, die im Wesentlichen neu oder neuartig und dem Produzenten zuvor unbekannt sind“ (Drevdahl et al. 1956). Allgemein wird Kreativität als die Fähigkeit angesehen, neue, unerwartete Artefakte zu produzieren. Weitere relevante Eigenschaften sind zudem Nützlichkeit, Wert, Originalität und Effektivität der Lösung. Das 4‑P-Modell der angewandten Kreativität von Rhodes unterscheidet dazu vier Bestandteile der Kreativität: (1) Person mit ihren individuellen Fähigkeiten, (2) Prozess mit den Methoden des Problemlösens, (3) Press als die Umgebung und Rahmenbedingungen, (4) Produkt als Ergebnis des Prozesses (Rhodes 1961).

*Kreativitätstechniken* sind Verfahren, die den Kreativitätsprozess unterstützen und das kreative Denken anregen sollen. Die bekannteste Kreativitätstechnik stellt das Brainstorming dar. Über die Jahre wurden viele verschiedene Kreativitätstechniken entwickelt. VanGundy (1988) beschreibt in seinem Buch im Jahr 1988 bereits 105 Techniken, Luther (2013) spricht im Jahr 2013 von 240 Ideenfindungstechniken und von mehr als 500 Techniken, wenn der gesamte Prozess betrachtet wird.

Kreativitätstechniken können sowohl von einer Person als auch von Gruppen eingesetzt werden, wobei die Mehrzahl der Techniken eher auf Gruppen ausgerichtet sind. Die meisten Kreativitätstechniken leben von der Unmittelbarkeit des persönlichen Aufeinandertreffens, der örtlich und physischen Interaktion, sie bauen geradezu darauf, wenn etwa einzelne Materialien und Objekte (z. B. Brainwriting 6‑3-5) von Person zu Person weitergereicht werden. Virtuellen Treffen fehlt diese physische Unmittelbarkeit und sie kann auch in Video basierten Kommunikationsformen kaum adäquat eingebracht oder ersetzt werden.

## Die Entwicklung einer Evaluationsmatrix für Kreativitätstechniken

Die Entwicklung der Evaluationsmatrix geschieht in einem mehrstufigen Prozess. Nähere Details zur Vorgehensweise und Methode finden sich in Kubla (2020). Im Folgenden werden die wesentlichen Schritte dieser Vorgehensweise dargestellt.

1. In einem ersten Schritt wurde als Basis für die Auswahl der Kreativitätstechniken deren Bekanntheitsgrad ermittelt. Eine häufige Nennung auf verschiedenen Internetseiten und in Büchern deutet auf eine hohe Bekanntheit hin. Für die Durchführung der Suche wurden die Begriffe „Kreativitätstechniken“, „Problemlösungstechniken“ und „kreative Problemlösungstechniken“ sowie die englische Übersetzung der Begriffe verwendet.
  Nach der Auszählung der Techniken wurden die Techniken, die unter verschiedenen Namen erwähnt wurden oder in deutscher und englischer Version vorkamen, aber die gleiche Technik darstellen, zusammengeführt und unter einem Namen zusammengefasst. Als Beispiel kann hier die Kombination der Kopfstandtechnik und der Umkehrtechnik genannt werden, da es sich hier um die gleiche Technik mit zwei unterschiedlichen Namen handelt.
  Von den 611 gefunden Techniken wurden alle ausgeschieden, die weniger als sechs Nennungen aufwiesen. Als Ergebnis blieben die in Tab. 1 aufgeführten 46 Techniken übrig. Einen Überblick über die die ermittelten Kreativitätstechniken gibt Kubla (2020). Siehe dazu auch Tab. 2 im Online Supplement.
2. In einem zweiten Schritt wurden die Merkmale ermittelt, mit Hilfe derer die einzelnen Kreativitätstechniken merkmalsbasiert und übersichtlich dargestellt werden sollen. Dies basiert einerseits auf in der Literatur bereits beschriebenen fünf Merkmalen (in Tab. 2 fett dargestellt), andererseits auf in weiteren Analysen ermittelten acht Merkmalen. So konnten insgesamt dreizehn Merkmale herausgearbeitet werden (Tab. 2). Für Details zur Ermittlung der Merkmale siehe Kubla (2020).
3. In einem dritten Schritt wurden für die einzelnen Merkmale Merkmalsausprägungen aus der Literatur ermittelt und in generelle Merkmalsausprägungen kondensiert. Am Beispiel des Merkmals Material wurden aus der Literatur folgende Merkmalsausprägungen ermittelt: Schreibmaterial, Visualisierungsfläche, Checklisten, Aufnahmegeräte etc. Diese wurden dann in die drei generellen Merkmalsausprägungen „Standard“, „Spezifisch“ und „Variabel“ eingeteilt.
4. In einem vierten Schritt wurde analysiert, wie essenziell die einzelnen Merkmale für die Auswahl einer Kreativitätstechnik sind. Merkmale mit der Priorität 1 sind dabei essenziell für die Auswahl einer Kreativitätstechnik, Merkmale der Priorität 2 spielen eine wichtige Rolle für die Auswahl einer Kreativitätstechnik und Merkmale der Priorität 3 haben nachgeordneten Einfluss auf die Auswahl. Die letzte Spalte enthält die Merkmale, die nur zusätzliche Informationen liefern, wie beispielsweise, ob das Ideenfindungsprinzip auf intuitiv-kreativer oder systematisch-analytischer Vorgehensweise beruht (siehe Tab. 2, 3 und 4).

**Tab. 1:** Die 46 ausgewählten Kreativitätstechniken sortiert nach der Anzahl der Erwähnungen *N* ≥ 6 in der Literatur (Basis: 10 Bücher, 90 Internetseiten mit insgesamt 611 Techniken)

Technik	*N*	Technik	*N*	Technik	*N*
Brainstorming	77	CNB	13	PMI	7
Mind Mapping	50	5 Why-Technik	12	PO	7
Sechs Denkhüte	38	SWOT	11	Reverse Brainstorming	7
Brainwriting 6‑3‑5	36	Attribute Listing	10	Reizbildanalyse	7
Morph. Analyse	32	Bisoziation	10	Visualisierung	7
Kopfstandtechnik	31	CPS	10	Action Plan	6
Brainwriting	27	Design Thinking	10	Brainwalking	6
Ishikawa Diagramm	27	KJ-Technik	10	Kartenumlauftechnik	6
Disney Denkstühle	27	ABC Wortliste	9	Delphi Technik	6
Osborn’s Checkliste	20	Semantische Intuition	9	Metapher Technik	6
Synektik	20	5W/5W1H-Technik	8	Pinnwandmoderation	6
TRIZ	17	Brainwriting-Pool	8	Problemlösungsbaum	6
SCAMPER	16	Clustering	8	Progressive Abstraktion	6
Analogie Technik	15	World Café	8	Relevanzbaumanalyse	6
Reizwortanalyse	14	Dotmocracy	7	Solo Brainstorming	6
Bionik	13

**Tab. 2:** Prioritäten der berücksichtigten Merkmale

Priorität 1 essenziell	Priorität 2 wichtig	Priorität 3 nachgeordnet	Priorität 4 Information
**Prozessphase**	Dauer	Gruppenteilung	**Prinzip**
**Einzel/Gruppe**	**Dynamik**	Moderator	**Art**
Schwierigkeit	Interaktion	Protokollant	
	Material	Teilnehmerzahl

**Tab. 3:** Merkmale mit Merkmalsausprägungen und dafür verwendete Abkürzungen (in englischer Sprache, da die Abkürzungen den englischen Begriffen entsprechen)

Merkmal	Abk	Merkmalsausprägungen	Abk	Merkmalsausprägungen
Process Phase	1 2 3 4	Problem Specification Idea Generation Idea Evaluation and Selection Idea Implementation	MP CP GA	Multiple Phases Cross-Phase General Approach
Individual/ Group	I IG	Individual Technique Individual & Group Technique	G G*	Group Technique Suitability for Large Groups
Difficulty	E M	Easy (Low Difficulty) Medium (Medium Difficulty)	H	Hard (High Difficulty)
Dynamics	SI LO MO	Silent Loud Moving	CB VA	Combination Variable
Interaction	NI VI PI	No Interaction Verbal Interaction Physical Interaction	CB VA	Combination Variable
Material	S SP	Standard Specific	VA	Variable
Duration	SD MD	Short Duration Medium Duration	LD VA	Long Duration Variable
Group Division	Y	Yes	N	No
Moderator	Y	Yes	N	No
Secretary	Y	Yes	N	No
Ideal Number of Participants	S (+/−)	Standard (with plus and/or minus → extra information for additional suitability for smaller and/or larger groups)	VA	Variable
Idea- Triggering Principle	IC SA CB	Intuitive-Creative Systematic-Analytic Combination	VA /	Variable No Idea Generation Technique
Type of Idea Generation	FAS SAS CBT	Technique of Free Association Technique of Structured Association Combination Technique	CFT IMT /	Confrontation Technique Imagination Technique No Idea Generation Technique

**Tab. 4:** Evaluationsmatrix für Kreativitätstechniken mit Merkmalen (Spalten 2–14) und Merkmalsausprägungen (Zellen). Die vollständige Tabelle ist im Online-Supplement Tab. 3 zu finden

Kreativitätstechnik	Prozess Phase	Einzel/Gruppe	Schwierigkeit	Dynamik	Interaktion	Material	Dauer	Gruppenteilung	Moderator	Protokollant	Teilnehmerzahl	Prinzip	Art
5 Why	1	IG	M	LO	VI	S	SD	N	Y	Y	S	x	x
Reizwort	2	IG	M	LO	VI	SP	MD	N	Y	Y	S +/−	IC	CFT
Brainwalking	2	G	M	MO	VI	S	SD	N	Y	N	S	IC	FAS
PMI	3	IG	M	LO	VI	S	SD	N	Y	Y	S	x	x
Action Plan	4	IG	E	LO	VI	S	SD	N	Y	N	S	x	x
Mind Map	MP	IG	E	VA si/lo	VA ni/vi	S	SD	N	Y	N	S +	CB	FAS
Disney	CP	IG	M	LO	VI	SP	VA	N	Y	Y	S +/−	CB	SAS
Design Thinking	GA	IG	H	VA	VA	VA	VA	VA	Y	Y	VA	VA	VA

## Das Entscheidungsmodell für den Einsatz von Kreativitätstechniken in der Online-Kommunikation

Basierend auf der Evaluationsmatrix wurde ein Entscheidungsmodell entwickelt, mit dem überprüft werden kann, ob eine Kreativitätstechnik für die Verwendung in der Online-Kommunikation geeignet ist. Hierfür wurden zunächst Anforderungen ermittelt, die für eine Eignung in der Online-Kommunikation erfüllt sein müssen. Dazu wurden die in Tab. 3 dargestellten Merkmale und Merkmalsausprägungen auf ihre Anwendbarkeit in der Online-Kommunikation analysiert.

Das Ergebnis bilden fünf Anforderungen.

- Anforderung 1: Es muss sich um eine einzelne Technik wie z. B. Brainstorming und nicht um einen allgemeinen Ansatz wie z. B. Design Thinking, handeln, da hier wiederum weitere Techniken Einsatz finden können.
- Anforderung 2: Eignung für Einzelpersonen/Gruppen. Individualtechniken, die nicht in Gruppen eingesetzt werden können, sind nicht für die Online-Kommunikation von mehreren Personen geeignet.
- Anforderung 3: Keine Bewegungsdynamik notwendig. Beispielsweise ist ein Ortswechsel innerhalb eines Raumes bei der Verwendung eines Computers nicht sinnvoll umsetzbar.
- Anforderung 4: Keine physische Interaktion notwendig. Physische Interaktion ist bei der Verwendung eines Computers nicht sinnvoll umsetzbar.
- Anforderung 5: Benötigte Materialien müssen für die Online-Kommunikation geeignet sein. Standardmaterial ist immer geeignet.

Um die geeigneten Techniken noch weiter differenzieren und bewerten zu können, wird eine Nutzwertanalyse (Zangemeister 2015) verwendet. Für die einzelnen Techniken wurden Nutzwerte berechnet. Als Kriterien für besondere Eignung wurden

- eine kurze Dauer (D),
- eine geringe Schwierigkeit (S),
- die Übertragbarkeit des Materials ohne Anpassungen (U) und
- keine Notwendigkeit einer Gruppenteilung (G)

identifiziert, da diese Merkmale eine Anwendbarkeit in der Online-Kommunikation nicht ausschließen, aber Einfluss darauf haben, wie gut eine Technik online angewendet werden kann. Alle vier Kriterien erhielten ein Gewicht von 25 %. Die Bewertung der einzelnen Kriterien erfolgt auf einer Skala von 1 bis 3, wobei der Wert 3 für sehr gute Eignung, der Wert 2 für gute und der Wert 1 für vergleichsweise weniger gute Eignung steht. Die Berechnung des Nutzwerts (N) erfolgt mit folgender Formel:

$$\mathrm{N}(\text{Kreativit"atstechnik})=\mathrm{D}\times 0,25+\mathrm{S}\times 0,25+\mathrm{U}\times 0,25+\mathrm{G}\times 0,25$$

**Abb. 1:**
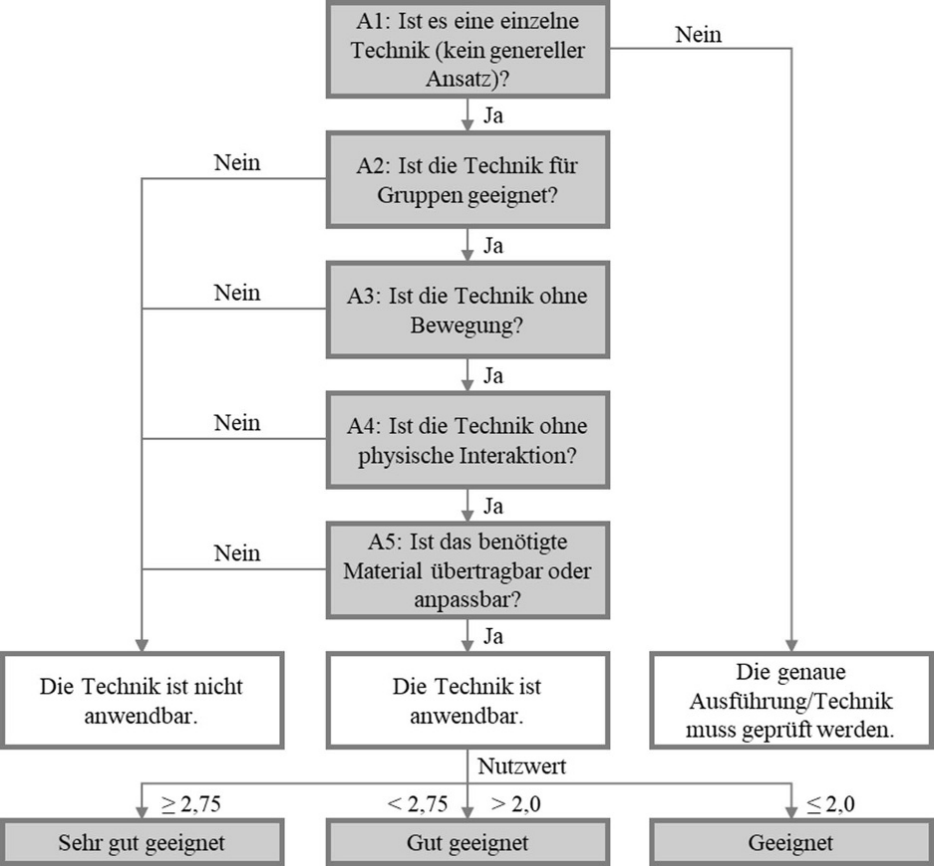
Entscheidungsbaum als Entscheidungsmodell

Basierend auf den Anforderungen und der Nutzwertanalyse wurde folgender Entscheidungsbaums als Entscheidungsmodell abgeleitet (Abb. 1).

Am Beispiel der Technik der Reizwortanalyse verläuft die Durchführung des Entscheidungsmodells wie folgt:

**Table:** 

A1 → JA	A2 → JA	A3 → JA	A4 → JA	A5 → JA

$$\text{Nutzwert}=3\times 0,25+3\times 0,25+2\times 0,25+2\times 0,25=\mathbf{2,5}$$

Das Ergebnis ist daher, dass die Technik *gut* geeignet ist.

Die Anwendung auf die Technik des Brainwalking sieht folgendermaßen aus:

**Table:** 

A1 → JA	A2 → JA	A3 → NEIN

Die Beantwortung der dritten Frage mit *Nein* liefert damit das Ergebnis, dass die Technik nicht anwendbar ist.

## Anwendung der Evaluationsmatrix auf die bekanntesten 46 Kreativitätstechniken

Für die Erstellung der Liste geeigneter Techniken wurden die 46 Kreativitätstechniken aus Tab. 1 anhand des im Kap. 6 erstellten Entscheidungsmodells untersucht. Von den 46 untersuchten Techniken konnten 34 Techniken als in der Online-Kommunikation anwendbar, neun Techniken als nicht anwendbar und drei Techniken als nicht direkt überprüfbar eingestuft werden, da es sich nicht um einzelne Techniken, sondern um generelle Ansätze oder Technikkombinationen handelt (s. Tab. 5).

## Anwendungsbeispiele bei der Entwicklung einer App

Die ModernSWApp Software GmbH muss aufgrund der COVID-Pandemie alle Mitarbeiter kurzfristig ins Home Office schicken. Unter Hochdruck arbeitet das Team Smartwatch an einem Kundenauftrag für eine App zur Erkennung des Blutdrucks mit Machine Learning.

### Fallbeispiel 1: Erstellen eines Flyers für die Blutdruck-App

Die Marketingabteilung der ModernSWApp GmbH möchte einen Flyer entwerfen, der für die App zur Erkennung des Blutdrucks die Einzigartigkeit der App in den Vordergrund stellt. Bis jetzt hat das Marketingteam dazu immer Brainwalking durchgeführt. Ist das Brainwalking für die Anwendung in der Online-Kommunikation geeignet und wenn nein, welche Technik könnte als Ersatz dienen?

Der Marketingleiter sieht aus Tab. 5, dass Brainwalking für die Anwendung im Home Office nicht geeignet ist. Er sucht als Alternative in Tab. 5 eine sehr gut geeignete Technik. Da er in anderen Projekten mit Brainstorming gute Erfahrungen gemacht hat, entscheidet er sich für Brainstorming, da auch die vorhandene Team-Software kollaborative Whiteboards unterstützt.

**Tab. 5:** Übersicht über die Eignung der Kreativitätstechniken für die Online-Kommunikation

Anwendbar (34)	Sehr gut geeignet	5 Why-Technik, 5W/5W1H-Technik, ABC Wortliste, Action Plan, Attribute Listing, Brainstorming, Clustering, CNB, Kopfstandtechnik, Metapher Technik, Mind Mapping, Pinnwandmoderation, PMI, SCAMPER
	Gut geeignet	Analogie Technik, Delphi Technik, Disney Denkstühle, Ishikawa Diagramm, Morphologische Analyse, Osborn’s Checkliste, PO, Problemlösungsbaum, Progressive Abstraktion, Reizwortanalyse, Reverse Brainstorming, Semantische Intuition, Sechs Denkhüte, SWOT, Reizbildanalyse
	Geeignet	Bionik, Bisoziation, Relevanzbaumanalyse, Synektik, World Café
Nicht anwendbar (9)		Brainwalking, Brainwriting, Brainwriting 6‑3‑5, Brainwriting Pool, Kartenumlauftechnik, Dotmocracy, KJ-Technik, Solo Brainstorming, Visualisierung
Weitere Prüfung (3)		CPS, Design Thinking, TRIZ

Die Brainstorming-Session wird vom Team mithilfe der üblichen Webkonferenzplattform durchgeführt. Ein Teammitglied nimmt die Rolle des Moderators und ein anderes Mitglied die des Schriftführers ein. Der Schriftführer sammelt dann alle geäußerten Ideen auf einem Online-Whiteboard oder in einem Präsentationsprogramm und macht die Ergebnisse durch die „Bildschirm Freigeben“-Funktion für alle sichtbar.

### Fallbeispiel 2: Einsatz in der Ideenfindung bei der Entwicklung der Blutdruck App

Am Tag nach der Home Office Entscheidung sollte sich das Entwicklerteam für eine Ideenfindungssitzung zur nicht invasiven Messung des Blutdrucks treffen. Im Zentrum steht die Frage, welche Sensoren die besten Daten liefern und wie verschiedene Sensoren optimal kombiniert werden können. Das Team hat bereits in anderen Projekten sehr erfolgreich Kreativitätstechniken eingesetzt und ist mit deren Grundprinzipien vertraut.

Das Team möchte diese Sitzung per Videokonferenz abhalten. Für die Auswahl der Kreativitätstechnik verwenden sie die Evaluationsmatrix und das Entscheidungsmodell. Zunächst müssen sie sich hierfür überlegen, welche grundlegenden Anforderungen erfüllt sein müssen. In diesem Fall muss die Technik der Phase der Ideengenerierung entsprechen und für Gruppen geeignet sein, die Schwierigkeit spielt keine Rolle. Durch die Eingrenzung weiterer Merkmale stehen am Ende zwei Techniken zur Verfügung. Für das Team kommen die Morphologische Analyse und Mind Mapping in Frage. Mithilfe der Tab. 5 überprüft das Team, ob diese für die Online-Kommunikation geeignet sind, und entscheidet sich letztendlich für die Morphologische Analyse.

Die Anwendung der Technik findet dann mithilfe der üblichen Webkonferenzplattform statt. Auch hier ist ein Mitglied des Teams der Moderator und ein anderes Mitglied der Schriftführer. Der Schriftführer sammelt dann alle geäußerten Ideen auf einem Online-Whiteboard oder in einem Präsentationsprogramm und macht die Ergebnisse durch die „Bildschirm Freigeben“-Funktion für alle sichtbar.

### Fallbeispiel 3: Prüfung auf Online-Kommunikationstauglichkeit einer neuen Kreativitätstechnik

Der Teamleiter ist in einer Ausgabe des Harvard Business Manager auf die Kreativitätstechnik „Rolestorming“ von Rick Griggs gestoßen (Griggs 2000). Beim Rolestorming wechseln die Teilnehmer ihre Perspektive in die einer anderen Person, wie beispielsweise des Kunden oder auch in die eines Superhelden, sodass sie über die Aufgabenstellung auf eine neue Art nachdenken müssen (VanGundy 2005).

Er möchte die Technik Rolestorming nun mithilfe des Entscheidungsmodells auf seine Anwendbarkeit in der Online-Kommunikation überprüfen. Nach näherem Studium der Technik ergeben sich die Ergebnisse bei Durchlaufen des Entscheidungsmodells wie folgt:

**Table:** 

A1 → JA	A2 → JA	A3 → JA	A4 → JA	A5 → JA

Durch die Beantwortung der Fragen kommt er zu dem Ergebnis, dass die Technik grundsätzlich in der Online-Kommunikation anwendbar ist. Anschließend berechnet er den Nutzwert, um zu erkennen, wie gut diese Technik geeignet ist.

$$\text{Nutzwert}=3\times 0,25+3\times 0,25+3\times 0,25+3\times 0,25=3$$

Damit eignet sich diese Technik sehr gut für den Einsatz im nächsten Meeting. Für die Durchführung verwendet das Team nun ihre übliche Webkonferenzplattform. Jedes Teammitglied wechselt anschließend in die jeweilig festgelegte Rolle. Die Ergebnisse werden von einer Person auf einem Whiteboard oder in einem Präsentationsprogramm gesammelt und durch die „Bildschirm Freigeben“-Funktion für alle sichtbar gemacht.

## Fazit und Ausblick

Es wurde ein Modell vorgestellt, das es ermöglicht, Kreativitätstechniken, die für die Anwendung in der Online-Kommunikation geeignet sind, zu ermitteln. Von den 46 berücksichtigten Techniken konnten 34 Techniken als geeignet für die Anwendung in der Online-Kommunikation von Remote-Teams identifiziert werden. Weiterhin konnten 14 der geeigneten Techniken als besonders gut und 15 Techniken als gut in der Online-Kommunikation anwendbar eingestuft werden. Darüber hinaus kann das entwickelte Modell zur Eignungsprüfung von noch nicht untersuchten oder neuen Kreativitätstechniken verwendet werden.

Die kontinuierliche Entwicklung der Möglichkeiten in der Online-Kommunikation wirkt sich auch auf die Anwendung von Kreativitätstechniken in der Online-Kommunikation aus. Durch immer neue Funktionen der Anwendungen für die Online-Kommunikation können immer mehr Kreativitätstechniken in der Online-Kommunikation angewendet werden, bis die Entwicklungen derart fortgeschritten sind, dass die Face-to-Face-Situation vollständig auf die Online-Situation übertragen werden kann.

Auch wenn eine Impfung ein relativ normales Arbeitsleben in der Pandemie ermöglicht, wird sich die Arbeitssituation im Unternehmen oder im Home Office stark verändern. Viele Unternehmen z. B. Google (Rixecker 2020) haben schon angekündigt, dass sie auch in Zukunft stärker auf Home Office setzen werden. Flexible Arbeitsplatzmodelle werden eher die Regel als die Ausnahme werden. Kostenersparnisse bei der Miete, Zeitgewinn für die Mitarbeiter durch Einsparung von Fahrten in das Büro, Mitarbeitergewinnung auch aus weiter entfernten Orten – diese Vorteile kommen vermehrt zum Tragen. Persönliche Treffen werden sich immer mehr in die Online-Welt verlagern. Dies gilt auch für den Einsatz von Kreativitätstechniken. Hier wird die Softwareindustrie gefordert sein, die geeigneten Techniken durch entsprechende Anwendungen zu unterstützen. Natürlich werden auch ortsgebundene Kreativitätstechniken weiter eine wichtige Rolle spielen, aber sie werden sukzessive durch ihre Online-Äquivalente ergänzt und teilweise ersetzt.

## Supplementary Information

Im Online Supplement zum Beitrag finden sich ausführliche Tabellen mit Beschreibungen zu den Kreativitätstechniken und die vollständige Evaluationsmatrix.
