# Supplementary material for: Impact of the Covid 19 Pandemic on the Use of Creativity Techniques: Challenges, Approaches, and Models for Online Communication in Virtual Teams
Source: HMD Prax Wirtsch Inform. 2021 Jun 28;58(4):816–29. [Article in German] doi: 10.1365/s40702-021-00752-w (PMC8237546; doi:10.1365/s40702-021-00752-w)
Supplement: Supplementary file 1 — Im Online Supplement zum Beitrag finden sich ausführliche Tabellen mit Beschreibungen zu den Kreativitätstechniken und die vollständige Evaluationsmatrix. [file 40702_2021_752_MOESM1_ESM.docx]

Online Supplement zu „Auswirkungen der Covid-19-Pandemie auf den Einsatz von Kreativitätstechniken: Herausforderungen, Lösungsansätze und Modelle für die Online-Kommunikation in Virtuellen Teams“

Die nachfolgenden Zusatzmaterialen wurden Kubla (2020) (im Literaturverzeichnis des Artikels zu finden) entnommen. Auf eine Übersetzung wurde größtenteils verzichtet. Für ein besseres Verständnis folgt nun eine Übersetzung der genannten Techniken ins Deutsche.

Tabelle 1: Übersetzung Techniken Englisch - Deutsch

| Englisch | Deutsch |
| --- | --- |
| Brainstorming | Brainstorming |
| Mind Mapping | Mind Mapping |
| Six Thinking Hats | Sechs Denkhüte |
| Brainwriting 6-3-5 | Brainwriting 6-3-5 |
| Morphological Analysis | Morphologische Analyse |
| Flip Flop Technique | Kopfstandtechnik |
| Brainwriting | Brainwriting |
| Ishikawa Diagram | Ishikawa Diagramm |
| Disney Thinking Chairs | Disney Denkstühle |
| Osborn's Checklist | Osborn's Checkliste |
| Synectics | Synektik |
| TRIZ | TRIZ |
| SCAMPER | SCAMPER |
| Analogy Technique | Analogie Technik |
| Random Words | Reizwortanalyse |
| Bionics | Bionik |
| CNB (Collective Notebook) | CNB (Collective Notebook) |
| 5 Why-Technique | 5 Why-Technik |
| SWOT | SWOT |
| Attribute Listing | Attribute Listing |
| Bisociation | Bisoziation |
| CPS (Creative Problem Solving) | CPS |
| Design Thinking | Design Thinking |
| KJ-Technique/Affinity Diagrams | KJ-Technik |
| ABC Word List | ABC Wortliste |
| Semantic Intuition | Semantische Intuition |
| 5W/5W1H-Technique | 5W/5W1H-Technik |
| Brainwriting-Pool | Brainwriting-Pool |
| Clustering | Clustering |
| World Café | World Café |
| Dotmocracy | Dotmocracy |
| PMI (Plus Minus Interesting) | PMI |
| PO (Provocative Operation) | PO |
| Reverse Brainstorming | Reverse Brainstorming |
| Visual Synectics | Reizbildanalyse |
| Visualization | Visualisierung |
| Action Plan | Action Plan |
| Brainwalking | Brainwalking |
| Circulating Cards Technique | Kartenumlauftechnik |
| Delphi Technique | Delphi Technik |
| Metaphorical Thinking | Metapher Technik |
| Pinboard Moderation | Pinnwandmoderation |
| Problem Solving Tree | Problemlösungsbaum |
| Progressive Abstraction | Progressive Abstraktion |
| Relevance Tree Analysis | Relevanzbaumanalyse |
| Solo Brainstorming | Solo Brainstorming |

Tabelle 2: Beschreibung der Kreativitätstechniken (Englisch)

| 5 Why-Technique | Starting with a why question related to the problem, further why questions are asked in relation to the previous answer, which redefines the problem (VanGundy, 1988, pp. 52-53). |
| --- | --- |
| 5W/5W1H-Technique | To gather information about the problem, the questions What, Where, When, Who, Why and How are answered in relation to the problem (VanGundy, 1988, pp. 46-47). |
| ABC Word List | An idea should be collected for each letter of the alphabet. Each participant can either create his or her own list or the participants can create the list together. (Luther, 2013, p. 199) |
| Action Plan | All necessary implementation measures of an idea or concept are collected and structured in terms of time. The presentation is done in a table with the columns what, who, until when and a column for success control. (Luther, 2013, p. 317) |
| Analogy Technique | At first, analogies to the problem definition are tried to be found, for which, for example, the question “What can be compared with this situation?” can be used. Then an analogy is selected and pursued by trying to find solutions for the analogy situation. As a last step these solutions are applied to the original problem. (Luther, 2013, p. 226) |
| Attribute  Listing | An object or process is first broken down into its individual characteristics and the current versions of these characteristics are described. Subsequently, variation possibilities are collected for each characteristic. The description is given in a table. (Schlicksupp, 2004, p. 90) |
| Bionics | The central principle is first determined from the task, to which analogies in nature are then collected. Subsequently the solutions of nature are analyzed and transferred to the original problem. (Brunner, 2008, pp. 116-117) |

| Bisociation | First, any image, object or other stimulus that is as far away as possible from the task is selected, which is then analyzed and described as precisely as possible. Subsequently, principles or commonalities are searched for that can be transferred to the problem, so that new approaches to the solution can be developed. (Luther, 2013, p. 259) |
| --- | --- |
| Brainstorming | The participants of a group express their ideas, suggestions and proposals concerning a task aloud, thus providing mutual impulses for each other. Criticism is prohibited and all contributions are allowed, as quantity is more important than quality. The statements of the participants are noted down by a secretary. (Brunner, 2008, pp. 128-129) |
| Brainwalking | Several flipcharts or pin boards are set up in a room with the same question or with different questions. Afterwards, the participants walk all over the room and write their ideas on the different posters. (Luther, 2013, p. 211) |
| Brainwriting | The participants sit at a table and write down their ideas, using a new sheet for each idea. Afterwards the sheets are passed on in the same direction and edited by the other participants until the sheet is back at the creator. (Luther, 2013, p. 194) |
| Brainwriting  6-3-5 | Six participants sit at a table and each have a sheet of paper with three columns and several rows in front of them, on which they write down three ideas each in 5-minute sections. Between each round, the sheets of paper are passed on in the same direction so that the other rows can be filled with new, complementary and varying ideas, inspired by the ideas of the previous one. (Schlicksupp, 2004, pp. 116-117) |
| Brainwriting-Pool | All participants sit at a table, which already has one or two sheets with three or four possible ideas in the middle. The participants then write all the ideas they can think of on one sheet. If they need new ideas, they can swap their sheet several times with a sheet from the middle of the table. Complementary or expanding ideas are written on the foreign sheet, for new ideas a new sheet is used. (Brunner, 2008, pp. 139-140) |
| Circulating Cards  Technique | The participants sit at a table and write down their ideas on cards, using a new card for each idea. After writing on a card, the participants always pass it on in the same direction so that the following persons can be inspired by this idea, whereby no more writing is allowed on the rotating cards. Cards that return to the creator are placed in the middle of the table, from where they are pinned to a pinboard so that the ideas can be explained, if necessary, and grouped. (Brunner, 2008, pp. 194-195) |
| Clustering | The task is written in the middle of a sheet. From this, associations are formed, each of which is the center for further associations. (Luther, 2013, pp. 192-193) |
| CNB  (Collective Notebook) | Each participant receives a notebook in which the question and relevant information are noted down. Within the agreed time, e.g. four weeks, the participants write down all thoughts about the question in the notebook. At the end of this period, the notebooks are collected and the ideas are evaluated. (Brunner, 2008, pp. 204-205) |
| CPS  (Creative  Problem  Solving) | CPS is a general approach to problem solving, consisting of the three phases explore the challenge, generate ideas, and prepare the action, whereby these are divided into further sub-steps. Other techniques, such as brainstorming, can be used in the phases. The phases can either be run through as a whole process or separately. (Luther, 2013, pp. 376-379) |
| Delphi  Technique | First, experts are selected, contacted and the questionnaire is created and sent out. The answered questionnaires are evaluated and analyzed. If the previously defined goal, such as consensus building, has been achieved, the Delphi survey is finished, and the results are statistically processed and published. If not, further rounds of questioning with evaluation of the answers are carried out, whereby the participants receive feedback, i.e. the results of the previous round, before each round. (Häder, 2014, pp. 91-92) |

| Design  Thinking | Design Thinking is an approach to problem solving in which user wishes and needs and user-oriented invention are the focal points. The success of Design Thinking is determined by a collaborative working and thinking culture consisting of multidisciplinary teams, a variable space, and the Design Thinking process. The process consists of several iterative steps, whereby the number of steps varies in the literature. (Hilbrecht and Kempkens, 2013, pp. 351-355) |
| --- | --- |
| Disney  Thinking Chairs | One after the other, three different roles are taken on, separated from each other by different stations or rooms. There is a short break between the individual roles. It begins with the role of the dreamer, in which all ideas are collected. This is followed by the role of the realist, in which the ideas are considered in terms of how they can be implemented. The last role is that of the critic, in which objections are developed. This sequence can be run through several times. (Luther, 2013, pp. 349-350) |
| Dotmocracy | There are different ideas to choose from, which the participants of a group evaluate using sticking points. All participants receive the same number of sticking points, depending on the number of participants and ideas, and an agreement is made on how many points may be awarded per idea. The ideas that receive the most points can be seen as the most popular ideas. (Luther, 2013, p. 289) |
| Flip Flop  Technique | At first, the question is turned upside down or reversed and ideas for the reversed question are collected. Subsequently, the ideas found are reversed again, i.e. transformed into the opposite. (Brunner, 2008, p 153) |

| Ishikawa  Diagram | First, a diagram in fishbone design is created, consisting of a horizontal arrow pointing to the right and four diagonal arrows pointing to this arrow, with the head of arrow also pointing to the right. The problem is noted at the tip of the horizontal arrow. On the other arrows are the labels Man, Machine, Method and Material. The participants then collect problem causes for each of the four areas and note them at the appropriate place in the diagram, thereby specifying the problem. (Luther, 2013, pp. 130-131) |
| --- | --- |
| KJ-Technique/  Affinity  Diagram | The first step is to collect as much information as possible on the problem, which is then written on separate cards and spread out on a surface. Subsequently, an attempt is made to combine cards whose information contents are related to each other, for which new cards with suitable generic terms are then created. The same procedure is used for the generic terms. If no further groupings are possible, the remaining generic terms are examined for their relationships and dependencies, further deepened and concretized by using individual cards if necessary. (Schlicksupp, 2004, pp. 75-77) |
| Metaphor  Technique | First, various metaphors related to the task are collected. Afterwards they are used as a starting point for finding new ideas, by investigating which solution approaches result from this for the task. (VanGundy, 1988, pp. 106-107) |
| Mind  Mapping | First the task is written in the middle of a sheet. Now main keywords and areas are collected, which lead away from this center as main branches. Keywords to the respective main branches leave the main branches as sub-branches. Further details can be attached to the sub-branches as twigs. (Luther, 2013, pp. 191) |

| Morphological  Analysis | Due to the summary of different terms there are two descriptions here:   1. Firstly, the main element of the task is broken down into its essential components, which are then written one below the other in the first column of a table. For each element, ideas are then collected and entered in the corresponding columns of the element row. To get a solution combination, one idea is selected from each row, which then together form the solution combination. This step can be repeated several times to get different solution combinations. (Schlicksupp, 2004, pp. 78-83) 2. First, the aspects by which the problem can be structured and broken down into its components are determined, whereby it is advisable to limit it to the two essential parameters. In case of two parameters, these aspects are entered as zero fields. Then the different values of the aspects are collected and entered in the first row respectively column of the table. Afterwards, the fields resulting from the combination of row and column are marked, which contain already known solutions or interesting combinations. (Schlicksupp, 2004, pp. 93-95) |
| --- | --- |
| Osborn’s Checklist | The questions of the Osborn checklist are answered in terms of task formulation. Within the checklist there are several spur questions on the aspects put to other uses, adapt, modify, magnify, minify, and substitute. (Brunner, 2008, pp. 269-273) |
| Pinboard  Moderation | All participants write down their ideas on individual cards, which are continuously collected by the moderator and pinned to a pinboard in an unorganized way, so that the ideas of the other participants can provide new inspiration. After finishing the idea finding process, the ideas are gone through and, if necessary, explained by the respective creators. If new ideas are found, they are written on new cards and pinned to pinboard. Afterwards the cards can be sorted forming clusters. (Schlicksupp, 2004, pp. 114-115) |

| PMI  (Plus, Minus,  Interesting) | An idea is examined for three aspects in order to determine the strengths and weaknesses. For this purpose, first the positive, then the negative and finally the interesting aspects of an idea are collected, whereby the criteria positions should be clearly separated from each other. (Luther, 2013, p. 301) |
| --- | --- |
| PO  (Provocative  Operation) | Initially, basic statements on the task are collected, which are then provocatively supplemented or alienated and, in addition, are given the initials PO at the beginning of the sentence. For the formulation of the PO statements, reversals, coincidences, or exaggerations can be used. Thereupon an attempt is made to collect ideas from the provocations, which are then transferred to the original question. (Luther, 2013, pp. 268-269) |
| Problem  Solving  Tree | A problem is noted at the top of a sheet. The solution is then broken down into its constituent parts by means of branching, which is done using various criteria. After completion, the problem solution tree has a hierarchical branching structure and shows all alternatives. (Schlicksupp, 2004, pp. 97-99) |
| Progressive Abstraction | The task is further and further abstracted by the repeated question of “What does it actually depend on?” (Schlicksupp, 2004, pp. 64-65). |
| Random Words | In a first step, one or more emotive words are selected from a list of emotive words that have nothing to do with the task. Afterwards, associations are collected with respect to the emotive words, which are then transferred to the original task. (Luther, 2013, p. 229) |
| Relevance  Tree Analysis | The procedure is identical to the problem solution tree, except that the alternatives have ratings added. (Schlicksupp, 2004, p. 99) |
| Reverse  Brainstorming | Due to the summary of different terms there are two descriptions here:   1. In the first step all weak points of the current solution of the problem are collected, whereupon in the second step improvement ideas are collected (Luther, 2013, p. 180). 2. Previously collected ideas are examined one after the other for their weaknesses. Then for each point of criticism an attempt is made to collect positive arguments that strengthen the idea or deal with the weaknesses so that the idea with the least weaknesses can be identified and selected. (Luther, 2013, p. 305) |
| SCAMPER | The questions of the Osborn checklist are answered in terms of task formulation (Brunner, 2008, pp. 270-274):   - Substitute: What can be substituted? - Combine: What can be combined? - Adjust: How can it be adjusted to the changed conditions? - Modify/ Magnify/ Minify: What can be modified/ magnified/ minified? - Put to Other Uses: How else can it be used? - Eliminate: What is dispensable? - Reverse/ Arrange: What would be the opposite/ How can it be rearranged? |
| Semantic  Intuition | First, terms from one or more subject areas are collected based on the problem definition and then combined randomly. Afterwards, these terms are examined to see what ideas they offer for the task. (Schlicksupp, 2004, p. 141) |
| Six Thinking Hats | Different colored hats are worn one after another, each of which having a different role. Alternatively, other colored objects can be used to replace the different hats.   - White = Information, Facts 🡪 neutral - Yellow = Advantages, Possibilities 🡪 optimistic - Black = Risks, Dangers, Concerns 🡪 critical - Green = Ideas, Creative Alternatives 🡪 creative - Red = Feelings, Intuition 🡪 emotional - Blue = Organization, Moderation 🡪 organized   The order of the hats is not fixed, but each hat must be put on once. Furthermore all participants always wear the same hat. (Brunner, 2008, pp. 178-184) |
| Solo  Brainstorming | Working individually, a question is thought through by expressing and noting all ideas and thoughts. The use of a recording device is useful for collecting thoughts without interruption. (Luther, 2013, p. 174) |
| SWOT | Different ideas or suggestions found are examined on four aspects. The strengths and weaknesses are addressed first, before the possibilities and obstacles are analyzed. (Luther, 2013, p. 304) |
| Synectics | The procedure is carried out in ten steps, whereby repeats or omissions of individual steps is possible. First, the problem is defined, and spontaneous solutions are collected, whereupon the problem is reformulated. In the following steps four to seven, different analogies are formed, of which one is selected in each step, on which the subsequent analogy is then based, so that a strong alienation from the problem is achieved. In steps eight and nine, the analogies found in step seven are then analyzed and applied to the problem. In the tenth step, solution approaches are developed. (Schlicksupp, 2004, pp. 131-136) |
| TRIZ | TRIZ offers various instruments, tools and principles for inventive problem solving that can be used for analysis, abstraction, idea generation, specialization and evaluation (Luther, 2013, pp. 387-389). |
| Visual  Synectics | First, different images are shown, which are analyzed one after the other. Thereby it is tried to find analogies to each image, which are then transferred to the task. (Luther, 2013, p. 260) |
| Visualization | First, relaxation exercises, such as muscle tension and relaxation, and warm-up exercises, such as visualizing and describing various images, are performed. Then the visualization of the task is done, describing all the things that can be seen in detail, why a recording device is useful. (VanGundy, 1988, pp. 97-98) |
| World Café | At first, all participants sit in smaller table groups in the room and discuss at the different tables under a common main topic at separate subtopics. After a set time, the participants move to any new table where another topic is discussed. Each table has a fixed table moderator who leads the respective discussion and keeps a table protocol. Furthermore, the tables are equipped with tablecloths that can be written on, so that the groups can record their thoughts in writing or by drawing. (Luther, 2013, pp. 399-400) |

Literatur

Brunner, A. (2008) Kreativer denken: Konzepte und Methoden von A-Z. (Lehr- und Studienbuchreihe Schlüsselkompetenzen). München: Oldenbourg

Häder, M. (2014) Delphi-Befragungen: Ein Arbeitsbuch. 3rd edn. Wiesbaden: Springer VS

Hilbrecht, H. and Kempkens, O. (2013) ‘Design Thinking im Unternehmen – Herausforderung mit Mehrwert’, in Keuper, F. et al. (eds.) Digitalisierung und Innovation: Planung, Entstehung, Entwicklungsperspektiven. Wiesbaden: Springer Gabler, pp. 347–364

Luther, M. (2013) Das große Handbuch der Kreativitätsmethoden: Wie Sie in vierSchritten mit Pfiff undMethode Ihre Problemlösungskompetenz entwickeln undzum Ideen-Profi werden.(Edition Training aktuell). Bonn: managerSeminar

Schlicksupp, H. (2004) Innovation, Kreativität und Ideenfindung. 6th edn. Würzburg: Vogel

VanGundy, A.B. (1988) Techniques of structured problem solving. 2nd edn. New York: Van Nostrand Reinhold

Tabelle 3: Evaluationsmatrix

| Creativity Technique | Process  Phase | Individual/ Group | Difficulty | Dynamics | Interaction | Material | Duration | Group Division | Moderator | Secretary | Participants | Principle | Type |
| --- | --- | --- | --- | --- | --- | --- | --- | --- | --- | --- | --- | --- | --- |
| 5 Why-Technique | 1 | IG | M | LO | VI | S | SD | N | Y | Y | S | x | x |
| 5W/5W1H-Technique | 1 | IG | M | LO | VI | S | SD | N | Y | Y | S | x | x |
| KJ-Technique | 1 | IG | M | CB (lo+mo) | VI | SP | LD | N | Y | Y | S | SA | x |
| Progressive Abstraction | 1 | IG | H | LO | VI | S | MD | N | Y | Y | S | SA | x |
| Ishikawa  Diagram | 1 | IG | H | VA (si/lo) | VI | S | MD | N | Y | N | S | x | x |
| Solo  Brainstorming | 2 | I | E | LO | NI | SP | SD | N | N | Y | x | IC | FAS |
| Visualization | 2 | I | H | LO | NI | SP | SD | N | N | Y | x | IC | IMT |
| Brainwriting | 2 | IG | E | SI | PI | SP | SD | N | N | N | S | IC | FAS |
| Clustering | 2 | IG | E | VA (si/lo) | VA (ni/vi) | S | SD | N | Y | N | S | CB | FAS |
| Attribute  Listing | 2 | IG | E | VA (si/lo) | VA (ni/vi) | S | MD | N | Y | N | S | SA | CBT |
| Flip Flop Technique | 2 | IG | M | LO | VI | S | SD | N | Y | Y | S | IC | CFT |
| Metaphorical Thinking | 2 | IG | M | LO | VI | S | SD | N | Y | Y | S | IC | CFT |
| Analogy Technique | 2 | IG | M | LO | VI | S | MD | N | Y | Y | S | IC | CFT |
| Semantic  Intuition | 2 | IG | M | LO | VI | S | MD | N | Y | Y | S | IC | CFT |
| Random Words | 2 | IG | M | LO | VI | SP | MD | N | Y | Y | S (+/-) | IC | CFT |
| Visual  Synectics | 2 | IG | M | LO | VI | SP | LD | N | Y | Y | S (+/-) | IC | CFT |
| ABC Word List | 2 | IG | M | VA (si/lo) | VA (ni/vi) | S | SD | N | Y | N | S | SA | SAS |
| Morphological Analysis | 2 | IG | M | VA (si/lo) | VA (ni/vi) | S | MD | N | Y | N | S (-) | SA | CBT |
| SCAMPER | 2 | IG | M | VA (si/lo) | VA (ni/vi) | SP | SD | N | Y | N | S | SA | SAS |
| Osborn's Checklist | 2 | IG | M | VA (si/lo) | VA (ni/vi) | SP | MD | N | Y | N | S (+) | SA | SAS |
| PO | 2 | IG | H | LO | VI | S | MD | N | Y | Y | S | IC | CFT |
| Synectics | 2 | IG | H | LO | VI | S | LD | N | Y | Y | S | IC | CFT |
| Bionics | 2 | IG | H | LO | VI | SP | LD | N | Y | Y | S | IC | CFT |
| Bisociation | 2 | IG | H | LO | VI | SP | LD | N | Y | Y | S | IC | CFT |
| Brainwriting 6-3-5 | 2 | G | E | SI | PI | SP | SD | N | N | N | S | IC | FAS |
| Brainstorming | 2 | G | E | LO | VI | S | SD | N | Y | Y | S (+/-) | IC | FAS |
| Pinboard  Moderation | 2 | G | E | CB (si+lo) | CB (ni+vi) | SP | SD | N | Y | N | S (+/-) | IC | FAS |
| Circulating Cards | 2 | G | E | CB (si+lo) | CB (pi+vi) | SP | SD | N | Y | N | S | IC | FAS |
| Brainwriting-Pool | 2 | G | M | SI | PI | SP | SD | N | N | N | S | IC | FAS |
| Brainwalking | 2 | G | M | MO | VI | S | SD | N | Y | N | S | IC | FAS |
| CNB | 2 | G* | E | SI | NI | SP | VA | N | N | N | VA | IC | FAS |
| PMI | 3 | IG | M | LO | VI | S | SD | N | Y | Y | S | x | x |
| SWOT | 3 | IG | H | LO | VI | S | SD | N | Y | N | S | x | x |
| Dotmocracy | 3 | G | E | MO | NI | SP | SD | N | N | Y | S | x | x |
| Action Plan | 4 | IG | E | LO | VI | S | SD | N | Y | N | S | x | x |
| Mind  Mapping | MP (1, 2) | IG | E | VA (si/lo) | VA (ni/vi) | S | SD | N | Y | N | S (+) | CB | FAS |
| Problem  Solving Tree | MP (1, 2) | IG | M | VA (si/lo) | VA (ni/vi) | S | LD | N | Y | N | S | SA | CBT |
| Relevance Tree Analysis | MP (1, 2) | IG | H | VA (si/lO) | VA (ni/vi) | S | LD | N | Y | N | S | SA | CBT |
| Delphi  Technique | MP (1, 2) | G | H | SI | NI | SP | VA | N | Y | N | VA | SA | SAS |
| Reverse Brainstorming | MP (2, 3) | G | H | LO | VI | S | MD | N | Y | Y | S (+/-) | IC | FAS |
| World Café | MP | G* | M | LO | VI | SP | LD | Y | Y | Y | VA | VA | x |
| Six Thinking Hats | CP (1-3) | IG | M | LO | VI | SP | VA | N | Y | Y | S | CB | SAS |
| Disney Thinking Chairs | CP (2-3) | IG | M | LO | VI | SP | VA | N | Y | Y | S (+/-) | CB | SAS |
| CPS | GA | IG | H | VA | VA | VA | VA | VA | Y | Y | VA | VA | VA |
| Design  Thinking | GA | IG | H | VA | VA | VA | VA | VA | Y | Y | VA | VA | VA |
| TRIZ | GA | IG | H | VA | VA | VA | VA | VA | Y | Y | VA | VA | VA |
